# Simple methods to reduce major allergens Ara h 1 and Ana o 1/2 in peanut and cashew extracts

**DOI:** 10.1002/fsn3.491

**Published:** 2017-07-25

**Authors:** Si‐Yin Chung, Christopher P. Mattison, Casey C. Grimm, Shawndrika Reed

**Affiliations:** ^1^ United States Department of Agriculture Agricultural Research Service Southern Regional Research Center New Orleans LA USA

**Keywords:** allergens, cashew, extraction/separation, IgE binding, *p*‐aminobenzamidine, peanut

## Abstract

Whole peanut or cashew extracts are usually used in immunotherapy. Reducing major allergen(s) in the extracts may lessen their side effects. Three methods were evaluated to reduce major allergens in peanut extracts: (1) *p*‐aminobenzamidine; (2) magnetic agarose beads; and (3) extraction of a commercial peanut flour at pH 7, respectively. The first two methods were also used to reduce major allergens in cashew extracts. After treatments, samples were evaluated by SDS‐PAGE. pABA‐treated samples were also analyzed for IgE binding in western blot. We found that the methods resulted in peanut extracts lacking detectable Ara h 1 but containing Ara h 2/6 and cashew extract lacking Ana o 1/2, but containing Ana o 3. Consequently, reduced IgE binding was observed. We conclude that the methods are useful for producing peanut or cashew extract with little Ara h 1 or Ana o 1/2.

## 1 INTRODUCTION

Peanut allergy is common and is a major public health concern. Peanut allergy is the most common cause of anaphylaxis in children and has become more prevalent in recent years. Strict peanut avoidance is the primary strategy to preventing anaphylaxis. However, such a strategy is almost impossible nowadays because many food products may contain food allergens in small amounts due to cross‐contamination caused using the same machinery for more than one food product. Peanut proteins may be undeclared or hidden in foods due to processing steps performed with the same machinery that processes peanuts or peanut products. Thousands of children are reported to visit the emergency rooms annually due to accidental ingestion of undeclared peanuts in foods (Hochstadter et al., 2016).

Various forms of immunotherapy are being investigated as treatment options. These include oral (i.e., introducing small doses of peanut proteins by mouth and gradually increasing the amount) (Tang & Hsiao, 2016), subcutaneous (i.e., peanut allergy shot) (Nelson, Makatsori, & Calderon, 2016), sublingual (i.e., peanut proteins in the form of drops introduced under the tongue) (Sindher, Fleischer, & Spergel, 2016), and epicutaneous immunotherapy (i.e., delivering peanut proteins through the skin via a patch on the skin) (Jones et al., 2016). They all have a common goal – to desensitizing peanut‐allergic individuals and induce the immune system to tolerate peanuts.

Despite some success, immunotherapy has limitations due to safety issues and risk of side effects. In many cases, people with severe peanut allergy cannot enroll in immuno‐therapy programs because whole peanut extracts containing the allergens are usually used. For instance, three of the above methods (i.e., subcutaneous, sublingual, and epicutaneous) involve using a whole peanut extract while in oral immunotherapy peanut flour is used. The high risk of adverse reactions associated with whole peanut extracts suggests a need for less allergenic peanut extracts for immunotherapy. Less allergenic peanut extracts may be beneficial because individuals with peanut allergy may be able to tolerate these extracts during immunotherapy.

In peanuts, Ara h 1 (63 kDa) and Ara h 2 (18‐20 kDa) are two major allergens that are recognized by more than 90% of peanut allergic patients (Montserrat et al., 2015). Modifying the allergens through chemical or physical means is reported to result in a less allergenic peanut extract. For instance, treatment of peanut extracts with oleic or tannic acid (Chung, Mattison, Reed, Wasserman, & Desormeaux, 2015; Chung & Reed, 2012) and pulsed UV light (a non‐thermal processing) (Chung, Yang, & Krishnamurthy, 2008; Zhao et al., 2014) have been shown to result in a decrease of IgE (immunoglobulin E) binding to Ara h 1 and Ara h 2. In one study, Ara h 2 has shown decreased binding to IgE when reduced and alkylated by dithiothreitol (DTT) (Apostolovic et al., 2013).

Here, we present three simple methods to produce peanut extracts lacking Ara h 1. These included: (1) *p*‐aminobenzamidine (pABA) to induce precipitation and removal of Ara h 1 from an extract; (2) magnetic 6% cross‐linked agarose beads to separate and remove Ara h 1 from an extract; and (3) extraction of commercial roasted peanut flour at pH 7 to obtain an extract lacking Ara h 1. pABA (in 1st method) is a protease inhibitor commonly used for purification of trypsin or serine proteases (Fasoli et al., 2013) and carries free amino groups which can be activated to cross‐link with and potentially induce precipitation of proteins (Evans, Olson, & Shores, 1982). Cross‐linked agarose beads (in second method) are spherical beads of gels commonly used in gel filtration or molecular size exclusion chromatography to fractionate proteins (He, Huang, Wang, Zhang, & Li., 2014). Several studies have shown that the agarose can selectively bind proteins, peptides, or polyphenols (Tan, Su, Gu, Xu, & Janson, 2010; Zhang et al., 2010). Lastly, extraction at pH 7 (in third method) was adopted because pH is reported to have an effect on extraction of proteins of different molecular weight or ionic charges (L'Hocine & Pitre, 2016). In this study, we presented peanut extract profiles that contained mostly Ara h 2 and a homologous allergen Ara h 6 (Ara h 2/6), but not Ara h 1 following treatments with the above individual methods. In addition, we determined if allergens (Ana o 1, 2, and 3) (Reitsma et al., 2016) from a cashew extract can be separated or removed in the same way.

## 2 MATERIALS AND METHODS

### 2.1 Materials

Para‐aminobenzamidine hydrochloride (pABA), glycine sodium salt and other L‐amino acids, 50% glutaraldehyde, trypsin, sodium borate decahydrate, Tris or phosphate buffered saline (TBS), Tween 20 (Tw 20), and EDC [1‐ethyl‐3(3‐dimethylaminopropyl)carbodiimide] were purchased from Sigma Co. (St. Louis, MO). Tris‐glycine pre‐cast gels (4%–20%), Novex mini‐cell apparatus for sodium dodecyl sulfate polyacrylamide gel electrophoresis (SDS‐PAGE), iBlot 7‐min blotting system, and bicinchoninic acid (BCA) protein assay kit were purchased from Thermo Fisher Scientific (Waltham, MA). NHS‐PEG‐COOH (N‐hydroxylsuccinimide‐polyethylene glycol carboxylic acid) was purchased from Nanocs. AquaStain was obtained from Bulldog Bio, Inc. (Portsmouth, NH). Monoclonal anti‐human IgE‐peroxidase was purchased from Southern Biotechnology (Birmingham, AL). Human sera from five individuals with peanut allergy (IgE levels determined by CAP‐FEIA = 70–100 kU/L) and six cashew allergic patients (IgE levels determined by CAP = 10‐65 kU/l) were obtained from PlasmaLab International (Everett, WA). Defatted roasted peanut meals were prepared from roasted Jumbo peanuts (Chung et al., 2015). Cashew nuts were purchased from nutsonline (https://nuts.com/). Amicon Ultra Centrifugal filters (0.5 ml, 10K) were purchased from Millipore (Billerica, MA). Magnetic 6% cross‐linked agarose beads (20–75 μm) were purchased from BioScience Beads (West Warwick, RI). Golden medium‐ and light‐roasted peanut flour were gifts from Golden Peanut and Tree Nuts (Alpharetta, GA).

### 2.2 Preparation of peanut and cashew extracts

Peanut extract was prepared according to the method of Chung et al. (2015). Briefly, defatted peanut meals (0.1 g) were stirred in 50 mmol/L sodium borate buffer (1 ml), pH 8.5 (for pABA reaction) or in 20 mmol/L Tris buffer, pH 7 (for magnetic beads) for 45 min at room temperature, and then centrifuged at 8,000 *g* for 5 min. The final extract was analyzed for protein concentration using the BCA assay. In a separate experiment, medium‐ or light‐roasted peanut flour (0.3 g) was extracted at various pH (3, 7.4, and 8.5) in citrate buffer, PBS, and 50 mmol/L borate buffer (1.5 ml), respectively, for 45 min and then centrifuged as described above. The protein concentration of each extract was also determined.

For the preparation of cashew extract, defatted cashew flour was re‐suspended (1:10 w/v) in Tris buffer (20 mmol/L Tris pH 8.0, 200 mmol/L NaCl, 1 mm EDTA, and 1 mmol/L PMSF) for 1 hr with vigorous mixing followed by sonication (3 × 15 s) with a 60 Sonic Dismembrator (Fisher Scientific, Pittsburgh, PA) at room temperature. The extract was centrifuged at 12,000*g* for 30 min at 4°C to remove particulates.

### 2.3 Treatment of peanut and cashew extracts with pABA or amino acids

Peanut or cashew extract (diluted in 50 mmol/L sodium borate buffer, pH 8.5) was mixed with an equal volume of pABA, followed by the addition of an aliquot of 50% glutaraldehyde. The final concentrations of the extract, pABA, and gultaraldehyde were, respectively, 0.5 mg/ml, 20 mmol/L, and 1%. The mixture was then stirred for 2 hr, after which precipitates were removed and the resulting extract was concentrated by centrifuging in a centrifugal filter unit (10 kD cut‐off) at 14,000*g* for 5 min, followed by adding PBS (400 μl) and repeating the concentration process to remove excess pABA. The final concentrate was retrieved by centrifuging at 1,000 *g* for 2 min. The protein concentration was then determined in a BCA assay. The protein recovery was determined as follows: % protein recovery = (total mg of proteins in concentrate) × 100/total mg of proteins in extract applied. The same experiment was repeated using an amino acid (glycine, phenyl‐alanine, or aspartic acid) instead of pABA. All treated and untreated samples were then subjected to SDS‐PAGE.

### 2.4 Pretreatment with PEG derivative and then with amino acid or pABA

Peanut extract (1 mg/ml, 1 ml) was incubated with polyethylene glycol (PEG) derivative, namely, NHS‐PEG‐COOH (N‐hydroxylsuccinimide‐PEG‐COOH) (50 mol/L) in PBS (pH 7.4) for 1 hr at room temperature and then centrifuged in a filtering unit to remove excess PEG derivative. The pH of the resulting extract was then adjusted to 6.5 before adding glycine or pABA (20 mmol/L). and carbodiimide (50 μmol/L). After 1 hr, the reaction mixture was diluted in PBS and concentrated by centrifuging in a filtering unit.

### 2.5 SDS‐PAGE and Western blot analyses of pABA‐treated peanut and cashew extracts

Peanut or cashew extracts (1 mg/ml, 5 μl) treated or untreated with pABA were applied to a 4%–20% gel according to the method of Chung and Reed (2015). After SDS‐PAGE, the gel was stained with an AquaStain solution or transferred to a PVDF membrane in an iBlot apparatus. IgE binding was determined as previously described (Chung et al., 2015) with a pooled plasma containing IgE antibodies against peanut or cashew allergens, followed by detection using a mouse anti‐human IgE‐peroxidase (for peanut) or biotinylated anti‐IgE (for cashew). IgE binding was visualized by reaction with 4‐chloro‐1‐naphthol (peanut) or IRdye‐680‐labeled streptavidin.

### 2.6 Treatment of peanut and cashew extracts with magnetic 6% cross‐linked agarose beads

Prior to treatment, magnetic beads (1 ml) were washed twice with 1 mol/L NaCL in 20 mmol/L Tris buffer, pH 7 (3 ml each time), and then 5× with the Tris buffer (3 ml each time). Peanut or cashew extract (1 mg/ml) (1 ml) was added, and the bead mixture was rotated for an hr at room temperature. The extract was then separated and removed from the beads using a magnetic device. The remaining beads were washed 7× with 20 mmol/L Tris buffer, pH 7 (3 ml each time) in order to remove residual extract. Sodium chloride (1 mol/L NaCl, 1 ml) was then added to the beads to release the bound proteins. The elution time was 5 min before using a magnetic device to separate the beads from the NaCL solution. The resulting solution was concentrated by centrifuging in a centrifugal filter (10 kD cut‐off) as described above. The sample was then subjected to SDS‐PAGE analysis.

### 2.7 Extraction of partially defatted peanut flour at various pH

Extracts from light‐ and medium‐roasted partially defatted peanut flours were prepared at different pH (3, 7.4, and 8.5) by stirring the flours (0.5 g) in 10 mmol/L citric‐phosphate buffer (pH 3), PBS (pH 7.4), or 50 mmol/L sodium borate buffer (pH 8.5) for 40 min and centrifuging at 8,000*g* for 10 min. The resulting extracts were then applied to SDS‐PAGE.

## 3 RESULTS

### 3.1 Effect of pABA on peanut and cashew allergens in extracts

**Figure 1:**
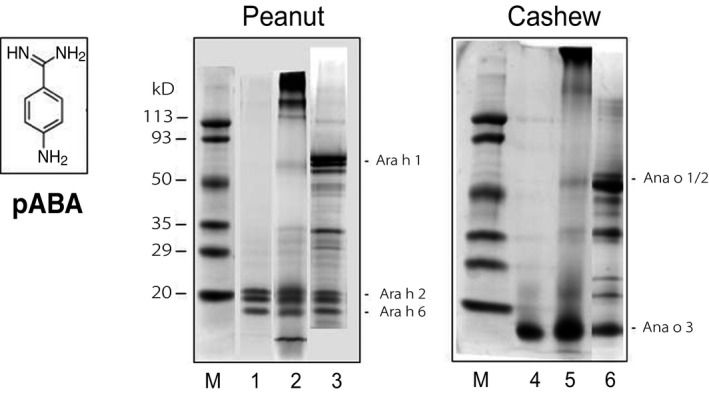
Structure of pABA and SDS‐PAGE profiles of peanut and cashew extracts treated with pABA or glycine in the presence of glutaraldehyde. M = marker; 1, 4 =  pABA‐treated; 2, 5 = glycine‐treated; and 3, 6 =  untreated

pABA has two free amino groups (Figure 1). Its effect on or cross‐linking with peanut allergens in the presence of glutaraldehyde was compared with that of glycine which has only one free amino acid. Results with peanut allergens showed that prior to treatment with pABA, peanut extract contains a number of proteins, including the allergens Ara h 1 (63 kDa), Ara h 2 (18‐20 kDa) and Ara h 6 (14 kDa) (Figure 1, lane 3). After treatment, the extract contained only Ara h 2 and Ara h 6 (Ara h 2/6), and not Ara h 1 or other proteins (lane 1). Ara h 1 and other proteins were not seen because they formed insoluble aggregates during the reaction and were removed from the extract prior to SDS‐PAGE. When glycine was used instead of pABA, no precipitates but several other soluble high molecular‐weight protein polymers (>135 kDa) were seen in the glycine‐treated extract (lane 2). Also, a small portion of Ara h 1 in addition to Ara h 2/Ara h 6 remained in the extract, indicating that the majority of Ara h 1 were converted to the soluble high molecular‐weight polymers. These polymers were thought to be aggregates of Ara h 1 because they were found to bind strongly to IgE antibodies (see IgE‐binding section below). In summary, treatment with pABA and centrifugation resulted in an extract lacking Ara h 1 but retaining Ara h 2/6. The protein recovery in this case was 16%.

We also determined the effect of pABA on cashew allergens. Figure 1 (SDS‐PAGE) shows the protein profiles of cashew extracts treated with and without pABA or glycine in the presence of glutaraldehyde. Untreated cashew extract contained three major allergens, namely, Ana o 1 (50 kDa), Ana o 2 (33 kDa), and Ana o 3 (13 kDa) (Figure 1, lane 6) while the pABA‐treated extract contained only Ana o 3 (lane 4). In comparison, the glycine‐treated extract contained soluble high molecular‐weight polymers (130 kDa) in addition to the three allergens (lane 5). The polymers were likely aggregates of allergens Ana o 1 and Ana o 2 since the polymers could be recognized by IgE antibodies (see IgE binding below).The finding indicates that a cashew extract containing only Ana o3 can be prepared using pABA. The protein recovery in this case was 22%.

### 3.2 Effect of phenylalanine and tyrosine on peanut allergens in extract

**Figure 2:**
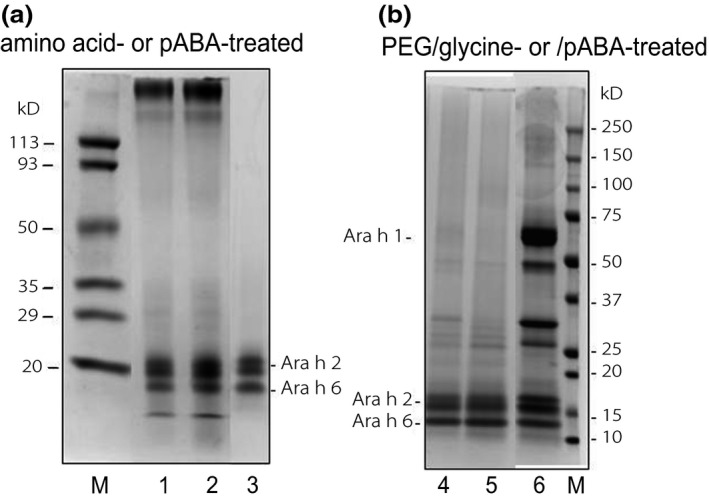
SDS‐PAGE profiles of peanut extract treated with amino acid, pABA, PEG/glycine, or PEG/pABA. 1 =  tyrosine‐treated; 2 =  phenylalanine‐treated; 3 = pABA‐treated; 4 =  PEG/glycine‐treated; 5 =  PEG/pABA‐treated; and 6 =  NHS‐PEG‐COOH‐treated (as control)

Because pABA possesses a benzene ring in addition to two amino groups, it is likely that the benzene ring in addition to the amino groups may also play a role in the precipitation of Ara h 1 from the peanut extract described above. We, therefore, determined if amino acids such as phenylalanine and tyrosine, which have a benzene ring, would induce Ara h 1 aggregation and precipitation from a peanut extract. We observed that unlike the pABA treatment (Figure 2a, lane 3), none of the amino acid treatments resulted in the precipitation of Ara h 1 from the extract (lanes 1‐2). Instead, the amino acid‐treated extracts (lanes 1‐2) contained soluble high molecular‐weight protein polymers (>113 kDa) in addition to Ara h 2/6 and a small amount of Ara h 1. As indicated above, the polymers were probably Ara h 1 aggregates, thus leaving a small amount of residual Ara h 1 monomers in the extract.

### 3.3 Effect of pretreatment with PEG derivative on peanut allergens in extracts

We demonstrated above that in contrast to pABA‐treated extracts, soluble high molecular‐weight polymers (>113 kDa) formed in glycine‐ or phenylalanine‐treated peanut extracts. This makes amino acids unsuitable for producing less allergenic peanut extracts without Ara h 1. To prevent formation of soluble polymers, we introduced a PEG (polyethylene glycol) derivative, namely, NHS‐PEG‐COOH, into the extract prior to treatment with glycine or pABA in the presence of carbodiimide. The function of NHS‐PEG‐COOH was to prevent cross‐linking between the allergens by blocking their free amino groups, thus leaving only free carboxyl groups available for activation by carbodiimide to cross‐link with the amino group(s) of pABA or glycine. Indeed, as shown in Figure 2b, high molecular‐weight protein polymers did not form after pretreatment of peanut extracts with the PEG derivative and then with glycine or pABA in the presence of carbodiimide (lanes 4‐5). In this case, the treated extracts contained mostly Ara h 2/6 without soluble polymers while the control (treated with PEG derivative but without pABA or glycine) contained both Ar a h 1 and Ara h 2 (lane 6).

### 3.4 IgE binding of pABA‐treated peanut and cashew allergens in western blot

**Figure 3:**
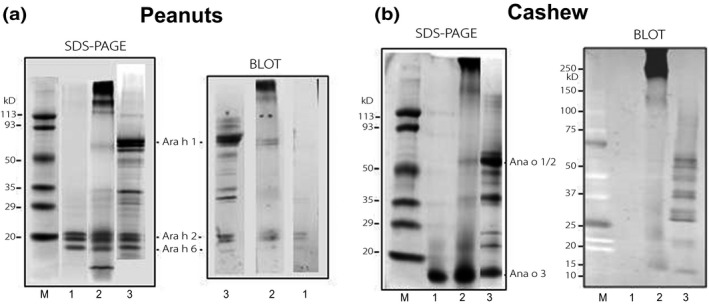
IgE binding in western blot assay of untreated and pABA‐ or glycine‐treated peanut and cashew allergens. SDS‐PAGE is also shown for comparison. 1 = pABA‐treated; 2 =  glycine‐treated; and 3 =  untreated

IgE binding to pABA‐treated nut extracts was evaluated by western blot to determine if there was a change in the allergenic capacity of the extract. We observed that after treatment with pABA, Ara h 2/6 exhibited a lower IgE binding (Blot, Figure 3a) (lane 3), compared to the untreated or glycine‐treated extract (Blot, lanes 1‐2). IgE binding to Ara h 1 was not detected in the pABA‐treated (Blot, lane 3) but was observed in the glycine‐treated extract (Blot, lane 2). High molecular‐weight polymers (>113 kDa) in the latter were strongly recognized by IgE antibodies, indicating that they were possibly aggregates of Ara h 1 since Ara h 1showed a reduced level as a monomer in SDS‐PAGE and blot (Figure 3a, lane 2).

IgE binding to cashew allergens was also analyzed after treatment with pABA. Results showed that despite appearing as a dark, thick band in SDS‐PAGE (Figure 3b, lane 1), Ana o 3 from the pABA‐ and glycine‐treated extract was either not recognized or weakly recognized by IgE antibodies (Blot, Figure 3b) (lanes 1‐2). However, polymers (>250 kDa) in the glycine‐treated extract were strongly recognized by IgE antibodies (Blot, lane 2), indicating that they may be aggregates of Ana o1 and/or Ana o 2. Overall, our findings indicate that IgE binding of Ara h 1 (peanut) and Ana o 1/2 (cashew) was reduced after treatment with pABA in the presence of glutaraldehyde.

### 3.5 Magnetic agarose beads to separate peanut or cashew allergens

**Figure 4:**
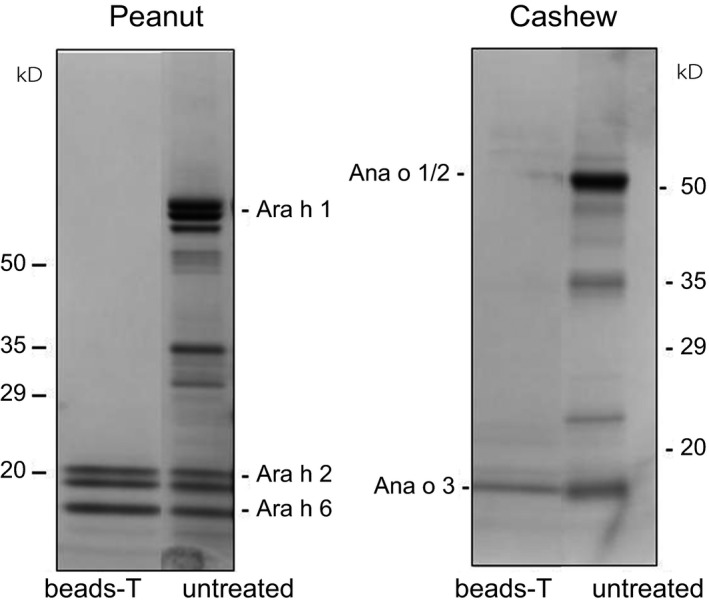
SDS‐PAGE profiles of untreated and magnetic beads‐treated peanut and cashew extracts. T  =  treated

Magnetic beads comprised of 6% cross‐linked agarose were used to isolate peanut or cashew allergens in extracts. Extracts were mixed with the beads, followed by washing and retrieving the bound proteins using 1 mol/L NaCl. We found that Ara h 2/6 from the peanut extract were the only proteins recovered by NaCl (Figure 4, peanut beads‐T). Although undetected in SDS‐PAGE, Arah 7/8 may exist in the fractions, but they could only be detected if specific antibodies were available.

The ability of NaCl to recover Ara h 2/6 from the beads suggests that the allergens bound to the beads through ionic interactions, and these interactions were disrupted by 1 mol/L NaCl, thus resulting in the release of Ara h 2/6 from the beads. The method was also applied to cashew extract, and we found that of the three major cashew allergens (Ana o 1, Ana o 2, and Ana o 3), only Ana o 3 was recovered by NaCl (Figure 4, cashew beads‐T). In all cases (i.e., peanut and cashew), there were no other proteins retrieved from the beads. This suggests that magnetic agarose beads could be used for removing Ara h 1 or Ana o 1/2 from peanut or cashew extract. The protein recoveries for Ara h 2/6 and Ana o 3 were 12% and 8%, respectively.

IgE binding was not performed on the isolated allergens because the indicated peanut and cashew allergens are consistent with the expected molecular masses in gel migration and besides, they did not undergo any changes or modifications following magnetic agarose beads treatment. The isolated allergens remained unmodified because the binding between the allergens and magnetic beads was mostly ionic interactions (see Discussion) which should not cause modifications to the allergens during or after isolation.

### 3.6 Effect of pH on extraction of partially defatted peanut flour

**Figure 5:**
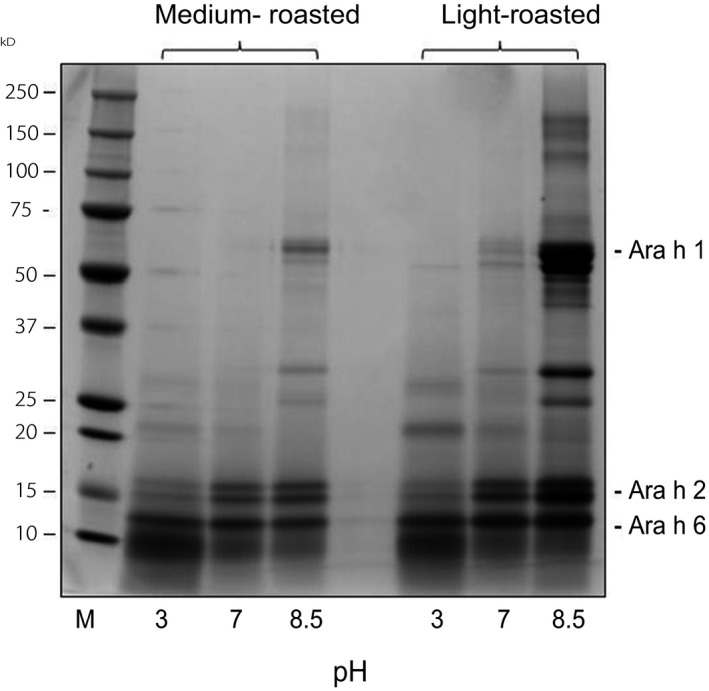
SDS‐PAGE profiles of peanut extracts obtained by extraction of partially defatted medium‐ or light‐roasted peanut flour at pH 3, 7, and 8.5

Peanut allergens (Ara h 1 and Ara h 2/6) generally can be extracted from defatted peanut meals at a pH 3 or above (Chung & Reed, 2012; Chung et al., 2015). However, this was not the case with the commercial medium‐roasted peanut flour. Results showed that when the medium‐roasted peanut flour was extracted at pH 7, Ara h 1 was missing and only Ara h 2/6 were seen in the extract (Figure 5, medium‐roasted). Extraction at pH 3 showed a similar result (i.e., Ara h 1 missing in extract), and in addition to that, the resulting extract contained much less of Ara h 2/6. All three allergens were eventually seen when extraction was performed at pH 8.5. Similar results were obtained with light‐roasted peanut flour with the exception that the extract at pH 7 contained a small amount of Ara h 1 (Figure 5, light‐roasted).

This latter finding indicates that this method (i.e., extraction at pH 7 to remove Ara h 1) may not be effective for all peanut flours and may only apply to the current commercial peanut flour. We evaluated this method on many roasted peanut meals and other peanut flours (commercially available), but none exhibited a lack of Ara h 1 after extraction at pH 7. Therefore, it is likely that it was the combination of the processing method of the selected peanut flour and the extraction (pH 7), which in some way made Ara h 1 harder to be extracted at pH 7.

## 4 DISCUSSION

Three methods (i.e., pABA, magnetic 6% cross‐linked agarose beads, and extraction at pH 7) have been described for the removal of peanut allergen Ara h 1 from a peanut extract. In the pABA method, Ara h 1 was precipitated because of the cross‐linking between pABA, glutaraldehyde, and Ara h 1. However, when glycine or other amino acid was used, there were no precipitates observed, but rather, soluble high molecular‐weight polymers were formed. The difference in formation of soluble high molecular‐weight polymers between pABA and glycine with regard to precipitating Ara h 1 is likely due to the presence of amino groups and benzyl group in pABA. The extra amino group in pABA (compared to one in glycine) probably constituted a major factor in the multiple linkage and consequent precipitation of Ara h 1.

On the other hand, the majority of Ara h 2/6 did not precipitate most likely because they are smaller in size and contain very few amino groups that were available for multiple cross‐linking between the allergens and pABA. Lehmann et al. (2006) has shown that Ara h 2/6 contain mostly nitrogen‐ and sulphur‐containing amino acids (e.g., glutamine, arginine and cysteine) that do not carry free amino groups. Despite this, Ara h 2 appeared to have been modified by pABA because IgE binding was reduced following pABA treatment. Similar observations were made with cashew allergens. That is, Ana o 1 and Ana o 2 were precipitated following pABA treatment, and the remaining, soluble allergen Ana o 3 showed a distinct reduction in IgE binding.

Another simple method to remove Ara h 1 from a peanut extract or to separate cashew allergens was by use of the magnetic 6% cross‐linked agarose beads. Cross‐linked agarose is generally used in gel filtration to separate and purify proteins based on their molecular size, and has been shown to bind and separate proteins through ionic interactions (Tan et al., 2010; Chudasam & Siddhanta, 2015). On this basis, we used magnetic beads to separate peanut or cashew allergens. The method was simple because the targeted proteins can be separated from the beads using a magnetic device. In this case, Ara h 1 was found to be separated from Ara h 2/6 by the magnetic beads. The binding between Ara h 2/6 and the beads appeared to occur through ionic interactions because Ara h 2/6 could be released from the beads by 1 mol/L NaCl whose function was to disrupt ionic interactions. Similarly, using 1 mol/L NaCl resulted in the recovery of cashew allergen Ana o 3 from the beads.

The third method involved extraction of a partially defatted commercial roasted peanut flour at pH 7 or 3 to obtain a peanut extract without Ara h 1 but with Ara h 2/6. Our results demonstrated that extraction at pH 7 or 3 resulted in medium‐roasted peanut extract lacking Ara h 1. In contrast, extraction at pH 8.5 led to the appearance of Ara h 1 in both extracts. The finding suggests that pH played a significant role in the extraction of Ara h 1 from this particular peanut flour. The matrix components in the flour may have played a role in our findings – that is, they may have bound to Ara h 1 via ionic interactions, and at pH 8.5 this ionic interaction was disrupted (like separation in ion exchange chromatography), thus releasing Ara h 1 from the matrix into the extract. It should be noted that this method (extraction at pH 7 or 3) to obtain a peanut extract lacking Ara h 1 applied only to the present commercial peanut flour. We observed that the method would not apply to roasted peanut meals or other peanut flours where the extracts contained both Ara h 1 and Ara h 2 (see Figure 1, lane 3).

Despite the lack of some proteins, the nutritive value of peanut or cashew extracts may not be a concern because as stated in the Introduction, extracts with reduced allergens are likely to be used in immunotherapy as an alternative to the whole extracts.

## 5 CONCLUSION

We describe three simple methods (i.e., pABA, magnetic beads, and extraction at pH 7) to produce peanut extract that lacked Ara h 1 but contained Ara h 2/6. Glycine could be used as a substitute for pABA to remove Ara h 1 only if a PEG derivative (i.e., NHS‐PEG‐COOH) was included. Extraction at pH 7 to produce a peanut extract without Ara h 1 applied only to the present commercial roasted peanut flour and not roasted peanut meals. The pABA and magnetic bead methods resulted in a cashew extract that contained only Ana o 3 and without Ana o 1 or 2.

## CONFLICT OF INTEREST

There is no conflict of interest to declare.

## References

[fsn3491-bib-0001] Apostolovic, D. , Luykx, D. , Warmenhoven, H. , Verbart, D. , Stanic‐Vucinic, D. , de Govardus A.H. Jong, G. A. H. , … Koppelman, S. J . (2013).Reduction and alkylation of peanut allergen isoforms Ara h 2 and Ara h 6; characterization of intermediate‐ and end products. Biochimica et Biophysica Acta (BBA) ‐ Proteins and Proteomics, 1834, 2832–2842. 24145103 10.1016/j.bbapap.2013.10.004

[fsn3491-bib-0101] Chudasam, N. A. , & Siddhanta, A. K. (2015). Facile synthesis of nano‐sized agarose based amino acid—Its pH‐dependent protein‐like behavior and interactions with bovine serum albumin. Carbohydrate Research, 417, 57–65. 26413976 10.1016/j.carres.2015.09.001

[fsn3491-bib-0002] Chung, S. Y. , Mattison, C. P. , Reed, S. , Wasserman, R. L. , & Desormeaux, W. A. (2015). Treatment with oleic acid reduces IgE binding to peanut and cashew allergens. Food Chemistry, 180, 295–300. 25766831 10.1016/j.foodchem.2015.02.056

[fsn3491-bib-0003] Chung, S. Y. , & Reed, S. (2012). Removing peanut allergens by tannic acid. Food Chemistry, 134, 1468–1473. 25005968 10.1016/j.foodchem.2012.03.057

[fsn3491-bib-0004] Chung, S. Y. , & Reed, S. (2015). IgE binding to peanut allergens is inhibited by combined D‐aspartic and D‐glutamic acids. Food Chemistry, 166, 248–253. 25053052 10.1016/j.foodchem.2014.06.004

[fsn3491-bib-0005] Chung, S. Y. , Yang, W. , & Krishnamurthy, K. (2008). Effects of pulsed UV‐light on peanut allergens in extracts and liquid peanut butter. Journal of Food Science, 73(5), C400–C404. 18576985 10.1111/j.1750-3841.2008.00784.x

[fsn3491-bib-0006] Evans, S. A. , Olson, S. T. , & Shores, J. D. (1982). p‐Arninobenzamidine as a fluorescent Probe for the active site of serine protease. Journal of Biological Chemistry, 257(6), 3014–3017. 7037776

[fsn3491-bib-0007] Fasoli, E. , Reyes, Y. R. , Guzman, O. M. , Rosado, A. , Cruz, V. R. , Borges, A. , … Bansa, V. (2013). Para‐aminobenzamidine linked regenerated cellulose membranes for plasminogen activator purification: Effect of spacer arm length and ligand density. Journal of Chromatography B, 930, 13–21. 10.1016/j.jchromb.2013.04.025 PMC3687526 23703544

[fsn3491-bib-0008] He, J. , Huang, M. , Wang, D. , Zhang, Z. , & Li, G . (2014). Magnetic separation techniques in sample preparation for biological analysis: A review. Journal of Pharmaceutical and Biomedical Analysis, 101, 84–101. 24809747 10.1016/j.jpba.2014.04.017

[fsn3491-bib-0009] Hochstadter, E. , Clarke, A. , Schryver, S. D. , LaVieille, S. , Alizadehfar, R. , Joseph, L. , … Ben Shoshan, M. (2016). Increasing visits for anaphylaxis and the benefits of early epinephrine administration: A 4‐year study at a pediatric emergency department in Montreal, Canada. Journal of Allergy and Clinical Immunology, 137(6), 1888–1890. 27106202 10.1016/j.jaci.2016.02.016

[fsn3491-bib-0010] Jones, S. , Agbotounou, W. K. , Fleischer, D. M. , Burks, W. A. , Pesek, R. D. , Harris, M. W. , … Benhamou, P. H. (2016). Safety of epicutaneous immunotherapy for the treatment of peanut allergy: A phase 1. Journal of Allergy and Clinical Immunology, 137(4), 1258–1261. 26920463 10.1016/j.jaci.2016.01.008

[fsn3491-bib-0011] Lehmann, K. , Schweimer, K. , Reese, G. , Randow, S. , Suhr, M. , Becker, W. M. , … Rosch, P. (2006). Structure and stability of 2S albumin‐type peanut allergens: Implications for the severity of peanut allergic reactions. Biochemical Journal, 395, 463–472. 16372900 10.1042/BJ20051728 PMC1462689

[fsn3491-bib-0012] L'Hocine, L. , & Pitre, M. (2016). Quantitative and qualitative optimization of allergen extraction from peanut and selected tree nuts. Part 2. Optimization of buffer and ionic strength using a full factorial experimental design. Food Chemistry, 194, 820–827. 26471623 10.1016/j.foodchem.2015.08.032

[fsn3491-bib-0013] Montserrat, M. , Sanz, D. , Juan, T. , Herrero, A. , Sanchez, L. , Calvo, M. , & Perez, M. D. (2015). Detection of peanut (Arachis hypogaea) allergens in processed foods by immunoassay: Influence of selected target protein and ELISA format applied. Food Control, 54, 300–307.

[fsn3491-bib-0014] Nelson, H. S. , Makatsori, M. , & Calderon, M. A. (2016). Subcutaneous immunotherapy and sublingual immunotherapy: Comparative Efficacy Current and Potential Indications, and Warnings—United States Versus Europe. Immunology and Allergy Clinics of North America, 36(1), 13–24. 26617224 10.1016/j.iac.2015.08.005

[fsn3491-bib-0015] Reitsma, M. , Bastiaan‐Net, S. , Sforza, S. , van der Valk, J. P. M. , van Gerth van Wijk, R. , Savelkoul, H. F. J. , … Wichers, H. J . (2016). Purification and Characterization of Anacardium occidentale (Cashew) Allergens Ana o 1, Ana o 2, and Ana o 3. Journal of Agricultural and Food Chemistry, 64, 1191−1201. 26769082 10.1021/acs.jafc.5b04401

[fsn3491-bib-0016] Sindher, S. , Fleischer, D. M. , & Spergel, J. M. (2016). Advances in the treatment of food allergy: Sublingual and epicutaneous immunotherapy. Immunology and Allergy Clinics of North America, 36(1), 39–54. 26617226 10.1016/j.iac.2015.08.008

[fsn3491-bib-0017] Tan, T. , Su, Z.‐G. , Gu, M. , Xu, J. , & Janson, J.‐C. (2010). Cross**‐**linked agarose for separation of low molecular weight natural products in hydrophilic interaction liquid chromatography. Biotechnology Journal, 5(5), 505–510. 20440717 10.1002/biot.201000017

[fsn3491-bib-0018] Tang, M. L. , & Hsiao, K. C. (2016). An update on oral immunotherapy for the treatment of food allergy. Paediatrics and Child Health, 26(7), 304–309.

[fsn3491-bib-0019] Zhang, X. , Samuelsson, J. , Janson, J. C. , Wang, C. , Su, Z. , Gu, M. , & Fornstedt, T. (2010). Investigation of the adsorption behavior of glycine peptides on 12% cross‐linked agarose gel media. Journal of Chromatography A, 1217, 1916–1925. 20167326 10.1016/j.chroma.2010.01.058

[fsn3491-bib-0020] Zhao, X. , Yang, W. , Chung, S. Y. , Sims, C. A. , Otwell, S. W. , & Rababah, T. M. (2014). Reduction of IgE immunoreactivity of whole peanut (*Arachis hypogaea* L.) after pulsed light illumination. *Food* . Bioprocess Technology, 7(9), 2637–2645.

